# Local Individual Preferences for Nest Materials in a Passerine Bird

**DOI:** 10.1371/journal.pone.0005104

**Published:** 2009-04-01

**Authors:** Adèle Mennerat, Philippe Perret, Marcel M. Lambrechts

**Affiliations:** 1 Centre d'Ecologie Fonctionnelle et Evolutive (UMR 5175), Montpellier, France; 2 Laboratoire Evolution & Diversité Biologique (UMR 5174), Toulouse, France; University of St. Andrews, United Kingdom

## Abstract

**Background:**

Variation in the behavioural repertoire of animals is acquired by learning in a range of animal species. In nest-building birds, the assemblage of nest materials in an appropriate structure is often typical of a bird genus or species. Yet plasticity in the selection of nest materials may be beneficial because the nature and abundance of nest materials vary across habitats. Such plasticity can be learned, either individually or socially. In Corsican populations of blue tits *Cyanistes caeruleus*, females regularly add in their nests fragments of several species of aromatic plants during the whole breeding period. The selected plants represent a small fraction of the species present in the environment and have positive effects on nestlings.

**Methodology/Principal Findings:**

We investigated spatiotemporal variations of this behaviour to test whether the aromatic plant species composition in nests depends on 1) plant availability in territories, 2) female experience or 3) female identity. Our results indicate that territory plays a very marginal role in the aromatic plant species composition of nests. Female experience is not related to a change in nest plant composition. Actually, this composition clearly depends on female identity, i.e. results from individual preferences which, furthermore, are repeatable both within and across years. A puzzling fact is the strong difference in plant species composition of nests across distinct study plots.

**Conclusions/Significance:**

This study demonstrates that plant species composition of nests results from individual preferences that are homogeneous within study plots. We propose several hypotheses to interpret this pattern of spatial variation before discussing them in the light of preliminary results. As a conclusion, we cannot exclude the possibility of social transmission of individual preferences for aromatic plants. This is an exciting perspective for further work in birds, where nest construction behaviour has classically been considered as a stereotypic behaviour.

## Introduction

Behavioural plasticity helps individuals to cope with variation in environmental conditions. Such variation in the behavioural repertoire of animals can arise from purely ecological causes (e.g. when local environmental conditions limiting the range of possible behaviours differ among populations), but may also be acquired by learning in a wide range of animal species. Many important behavioural traits can indeed be fine-tuned during life, either by individual learning (i.e. by trial and error) or by social learning (e.g. by imitating the behaviour of other individuals) (reviewed in [1]). Naïve young animals, in particular, may benefit from observing the behaviour of more experienced individuals by increasing the efficiency with which they perform crucial tasks such as finding food, avoiding predators or choosing a sexual partner, without paying the costs associated with individual learning [1], [2].

In vertebrates, many components of foraging and reproductive behaviours can be socially transmitted. For example, in Norway rats *Rattus norvergicus*, long-lasting food preferences are acquired after a few minutes interaction of a naïve individual with a demonstrator [3]. Young roof rats *Rattus rattus* learn from adults how to efficiently open pinecones [4]. Social learning of foraging routes has been demonstrated in female guppies [5], [6]. In birds, the most documented example of social learning is the acquisition of the song repertoire of songbirds by imitation of a tutor from the local population [7], [8]. Social transmission has been studied in a few other avian behavioural traits, most of them being observed on captive birds (e.g. feeding preferences [9], [10]; lid opening [11]; handling of new objects [12]). These laboratory studies are certainly useful in assessing the learning abilities of animals, disentangling genetic from environmental effects, controlling for confounding factors and testing predictions made from theoretical models (e.g. producer-scrounger models, reviewed in [13]). Yet they provide little information on the actual occurrence and ecological importance of social learning in wild populations (e.g. [14]).

Many bird species build nests that protect their eggs and chicks from climatic variation and predators. Nest building is closely linked to fitness (e.g. offspring survival) and is therefore under high selective pressure. The assemblage of nest materials in an appropriate structure is often typical of a bird genus or species, although within-species variation in the selection of nest materials can be significant [15], [16]. Such flexibility can be adaptive, e.g. when the nature and abundance of nest materials vary across habitats, and may be achieved through individual or social learning. In particular, new individuals in a population may gain efficiency from observing how other individuals find and select the proper nest materials and imitating them (e.g. [17]).

In addition to basic nest materials (e.g. moss or twigs), several bird species bring to their nests green plants which are rich in volatile secondary compounds [18], [19]. The selected plant species often represent a small fraction of the species available in the habitat [18], [20]. Several studies suggest that nest greenery is beneficial to chick growth, development or survival [21], [22].

On Corsica, hole-nesting female blue tits *Cyanistes caeruleus* regularly incorporate fresh fragments of several species of aromatic plants on the top of their nests (e.g. *Lavandula stoechas, Achillea ligustica, Helichrysum italicum*), and quickly replenish the nest with fresh fragments after experimental removal [23]–[25]. The maximal dry mass of plant fragments found in nests of blue tits is close to 1.3 g (Mennerat, pers. obs.). The plant species found in nests of blue tits represent only a small fraction of the plants species available in the habitat [24], and some of them possess *in vitro* antiseptic, fungicidal or insect-repellent properties [26], [27]. These aromatic plants reduce both the density and phylotypic richness of bacteria living on nestlings (Mennerat et al., unpublished data) and have positive effects on chick growth, feather development and hematocrit [28].

Strong inter-nest variation in aromatic plant species composition is frequently observed, but there are few quantitative data, and most arguments so far come from qualitative field observations (e.g. [23]). Here we used both comparative and plant-removal (cf [24]) approaches to explain spatiotemporal variations in the use of aromatic plants in blue tit nests from one valley in Corsica. We first tested whether the aromatic plant species composition in nests was individually repeatable, both within and across breeding seasons. Variation in the aromatic plant species composition of nests was then explored both across years and between territories within study sites to test the three following predictions.

First, the aromatic plant species composition in nests may depend on the presence of these plants in the surrounding territory. If true, we predicted inter-year similarity in nest composition for the same female to be lower between two breeding attempts in different nestboxes than between two breeding attempts in the same nestbox.

Second, breeding experience may affect the use of aromatic plants by females. For instance, yearling females may have less information on the aromatic plants available in their environment than experienced females, and therefore be less efficient at finding and bringing plants to their nests. If true, we predicted lower similarity in nest composition across two consecutive years when females passed from their first to second breeding attempt than for females who already had one previous breeding season prior to the start of the study.

Third, individual females may differ in their choices of aromatic plant species, i.e. there may be individual preferences for certain aromatic plant species (e.g. [29]). In that case, for a given nestbox, similarity in nest composition should be lower between two breeding attempts by different females than between two attempts by the same female.

Finally, to understand how these preferences differed at a larger spatial scale, we tested for differences in aromatic plant species composition of nests between distinct study sites (i.e. between distinct groups of adjacent territories).

## Results

### Individual repeatability of the aromatic plant species composition of nests

#### a) During the breeding season

The composition in aromatic plant species that female blue tits added in their nests within 24 h was repeatable across breeding stages. Aromatic plant composition was indeed significantly more similar within than among females (Anosim, N = 14, P = 0.002) and was not more similar within than across breeding stages (egg laying, incubation, chick rearing) (Anosim, N = 14, P = 0.31) (Table 1).

**Table 1:** Individual repeatability in aromatic plant species composition of blue tit nests, both across breeding stages and across years, as tested by analyses of similarity (Anosim, see Materials and Methods).

	Global R	P
Across breeding stages (N = 14 nests, 3 repeated measures per nest)		
Female (nest)	0.26	0.002 **
Breeding stage (egg laying, incubation or chick rearing)	0.01	0.31
Across years (N = 27 females)		
Female	0.54	<0.001***
Year (2005, 2006 or 2007)	<0.005	0.82

Low P-values mean that similarity within groups is significantly higher than between groups. Global R and P value are both calculated by the Primer v 6.1.6. software.

#### b) Across years

The composition of aromatic plant species that female blue tits added in their nests during one breeding season was repeatable across years. Inter-year similarity in aromatic plant species composition was indeed higher within than among females (Anosim, N = 27, P<0.001) and was not higher within than across years (Anosim, N = 27, P = 0.82) (Table 1).

### Spatial and individual factors of variation between nests

**Figure 1:**
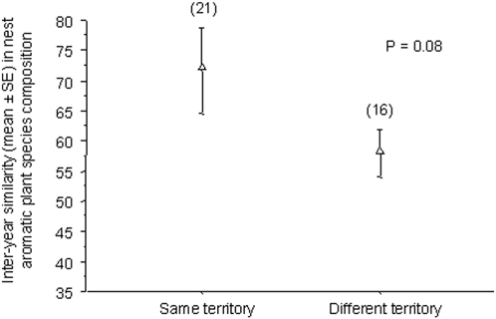
Similarity in the aromatic plant species composition of nests from breeding attempts made by the same female, either in the same territory or in different territories. Similarity was calculated using the Bray-Curtis index. Sample sizes are indicated in parentheses. The P-value results from a Student's t-test (see Methods).

Aromatic plant species composition of nests was only partly related to the territory. The similarity in plant species composition of nests between years was only marginally significantly lower for females that did not re-use the same nestbox than for females that did (t-test, d.f. = 34, P = 0.08) (Figure 1) (Table 2).

**Table 2:** Factors of variation in aromatic plant species composition of blue tit nests over successive years, as tested by t-tests comparing inter-year similarity in aromatic plant composition (see Materials and Methods).

	d.f.	t	P
Same female over successive years (N = 37 females)			
Change in territory	34	1.80	0.08 (*)
Acquisition of breeding experience	34	1.08	0.29
Same territory over successive years (N = 35 territories)			
Change in female identity	33	2.42	0.02*

d.f. = degrees of freedom, P = P-value.

Aromatic plant species composition of nests was not related to the acquisition of breeding experience. Inter-year similarity was not significantly lower for those females that passed from their first to their second breeding attempt (t-test, d.f. = 34, P = 0.29) than for more experienced females (Table 2).

**Figure 2:**
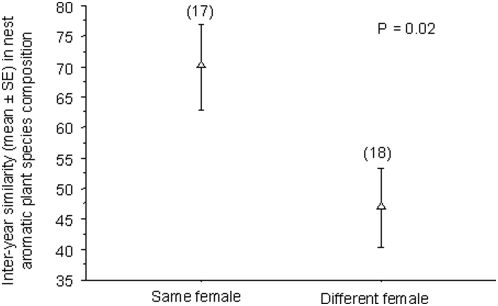
Similarity in the aromatic plant species composition of nests from breeding attempts made in the same territory, either by the same female or by different females. Similarity was calculated using the Bray-Curtis index. Sample sizes are indicated in parentheses. The P-value results from a Student's t-test (see Methods).

Finally, for a given nestbox, aromatic plant species composition varied according to female identity. The similarity in aromatic plant composition between two breeding attempts in the same nestbox was significantly higher when both attempts were made by the same female than when female identity differed (t-test, d.f. = 33, P = 0.02) (Figure 2) (Table 2).

### Inter-site variation in the aromatic plant species composition of nests

**Figure 3:**
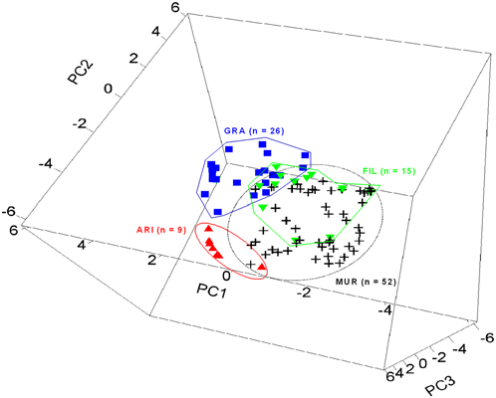
Three-dimensional representation of the variation in aromatic plant species composition of nests between study plots. Axes are those resulting from a PCA on the log-transformed relative abundances of the 15 aromatic plant species identified in nests (see Methods). Points representing nests from the ‘MUR’ site are located in the back of the figure, whereas points representing nests from the ‘FIL’ plot are in the front of it.

Aromatic plant species composition of nests strongly differed across study plots (Manova, N = 102, P = 0.0006), but not across years (Manova, N = 115, P = 0.70). The interaction between year and study plot was not significant (Manova, N = 115, P = 0.47) (Figure 3) (Table 3).

**Table 3:** Inter-site variability in aromatic plant species composition of blue tit nests, as tested by a Manova on the PC1, PC2 and PC3 scores from a Principal Component Analysis on relative abundances of aromatic species in 115 nests (see Materials and Methods).

	d.f.	Wilks	P
Year	2	0.89	0.70
Study site	4	0.36	0.0006***
Year×Study site	4	0.71	0.47

d.f. = degrees of freedom, Wilks = Wilks' lambda, P = P-value (as calculated by the R software).

## Discussion

This study provides new and unexpected evidence for individual preferences in the use of aromatic plants by blue tits. For a given individual female, the species composition of aromatic plants added in the nest varied little over time, both within a breeding season and across successive breeding attempts. In addition, for a given territory, the aromatic plant composition of nests varied according to female identity, which indicates that the nest aromatic plant composition results from individual preferences. The weak observed relation between changes in territory and changes in plant species composition of nests confirms that plant availability in the territory is only a marginal determinant of nest aromatic plant composition. This is consistent with previous findings that the presence of aromatic plant species in the territory is not significantly related to their presence in nestboxes [23]. The existence of individual preferences for aromatic plant species is also consistent with the observation that breeding blue tits are disturbed by an experimental change in the particular aromatic plant species composition of their nests [30]. Recent observations also indicate that individual olfactory preferences occur in another greenery-using species, the European starling [29].

A puzzling fact is the strong variation in plant species composition of nests across study plots, this composition being relatively homogeneous within study plots. In particular, there are striking differences in nest plant composition between the ‘ARI’ and ‘FIL’ plots and, to a lesser extent, between the three ‘ARI’, ‘FIL’ and ‘GRA’ plots located in the evergreen habitat (Figure 3, also see Figure S1). This is all the more surprising that these three plots are closely-located and ecologically very similar (see [31] for a detailed description). Several hypotheses may be proposed to explain such a pattern of variation.

First, as demonstrated in this study, the plant species composition of nests does not seem related to the precise location of nests (i.e. territory) within study plots. Yet nest composition could be related to the availability of plant species at the larger spatial scale of breeding plots. In that case, we would expect plants species to be added in nests in higher abundances in the plots where they are present, as compared to the plots where they are not found.

Second, individual olfactory preferences may arise from genetic and/or early environmental effects during the nestling period (e.g. [29]), i.e. they may be vertically transmitted from mother to daughter. If, additionally, females happened to be philopatric with respect to their plot of birth, then this would result in differences in nest composition across study plots.

Third, new females in a plot may reproduce the plant-adding behaviour of other females in this plot, e.g. by observing them when they collect and/or bring new plants into their nests. In other words, individual preferences for aromatic plants may be socially transmitted.

Preliminary data suggest that the observed differences in nest composition among plots do not match differences in the presence of aromatic plant species in the area covering these plots. In particular, some plant species are not found in nests although they are present in the study plot where these nests are located, whereas some plants are found in nests even if they are not found in this plot (see Table S1). Therefore, the relation between plant species composition of nests and plant availability in the environment does not seem strong enough to explain the observed variation in nest composition across study plots.

The hypothesis of a vertical transmission of individual preferences remains to be investigated in more details. This is an exciting perspective for future work, although several more years will be needed to estimate the mother-daughter heritability of nest plant composition. So far however, our data do not give any hint towards such a conclusion. In particular, females do not add plants in their nests in a composition similar to that added in the plot where they were born (see Figure S2). Additionally, dispersal between study plots frequently occurs, which excludes the possibility of a genetic differentiation between plots (see Table S2). Although being no definitive evidence against vertical transmission of individual preferences for aromatic plants, at least these preliminary results suggest that it is probably not the only mechanism underlying the strong spatial differences across study plots.

On the basis of these preliminary results, we therefore cannot exclude the hypothesis that individual preferences may be socially transmitted within study plots. Nests within plots are close to each other (30–40 m) in an open habitat [31]. Females thus have the opportunity to observe each other. Given that aromatic plants provide significant fitness benefits to blue tits [28], increased efficiency in finding and adding plants in nests should be selected for. Females arriving at a new breeding site might take advantage from acquiring information on the plant-adding behaviour of experienced neighbouring females. Such an imitation behaviour could contribute to the strong spatial variation in aromatic plant species composition of nests across study plots.

In a number of songbird species, social learning has been demonstrated in the context of the acquisition of song repertoire and results in local dialects [7], [8], [32]. Recent aviary experiments in another greenery-using species, the European starling *Sturnus vulgaris*, indicate that olfactory preferences can be acquired [29]. Our results show that there can be strong and consistent individual variation in nest-building behaviour in natural bird populations. More importantly, they do not exclude the possibility that social transmission of such behaviour might actually occur in wild populations of such a territorial small passerine bird. Further research on the modes of acquisition of nest-building behaviour in other passerine bird species may provide new insights into the potential for cultural transmission in birds.

## Materials and Methods

### Study sites and field protocols

The study was carried out during three consecutive years (2005–2007) in two distinct oak habitat types in Corsica where blue tits accept nestboxes for breeding (“Muro-deciduous”, 42°33′ N, 08°55′ E, broad-leaved deciduous oakwood, *Quercus humilis*; “Muro-evergreen”, 42°36′ N, 08°58′ E, evergreen oakwood, *Q. ilex*). The “Muro-evergreen” site is subdivided in three distinct plots (“ARI”, “FIL”, “GRA”) which are located at close distance from each other (approx.1 km, see [31], [33] for a detailed description of the sites). Juvenile dispersal is frequent between these three plots (see Table S2). All nestboxes were monitored throughout the breeding season to determine the onset of egg laying (March 1st = day 1), clutch size, hatching date, the number of hatchlings, and the number of chicks fledged. At days 14–15 post-hatching, all chicks were ring-marked. Both parents were captured and ring-marked, and their age (yearling or older bird) was assessed according to the colour of their primary wing coverts (more details on the field protocols can be found in e.g. [34]).

### Sampling and determination of aromatic plant fragments in nests

To investigate the spatial and temporal variation in amount and composition of aromatic plants, nests from the three study sites were collected at day 14–15 post-hatching, enclosed in hermetic plastic bags and replaced by the same amount of fresh moss. All nests were collected from first broods. To avoid damages caused by e.g. mites or micro-organisms before sampling of aromatic plant fragments, all nests were microwave-disinfected after collection (cf [25]). We then carefully inspected nests to separate aromatic plant fragments from the rest of the nest materials. Species determination was made from morphological characteristics, using a herbarium of local plants identified by a botanical specialist (A. Royaud) as reference. Aromatic plant fragments were then stored in paper bags and allowed to dry at ambient temperature for several weeks. Samples were finally weighed with a precision balance (Acculab Pocket Pro C/50) to the nearest 0.002 g to obtain the dry mass of each aromatic plant species per nest. All analyses of aromatic plant species composition presented here were performed using relative abundances (dry mass) of 15 plant species that could be identified with certainty (Table 4). Unidentified plant fragments represented 7.6%±1.4 SE of the total dry mass of plant fragments found in nests. Fragments identified as “*Mentha sp*.” were removed from the analyses because they may belong to several distinct species that are not easily distinguished from morphological characteristics (e.g. *Mentha suaveolens*, *Mentha aquatica*, *Calamintha nepeta*).

**Table 4:** Mean (±SD) relative abundances of fifteen plant species identified from blue tit nests.

	Mean (±SD) relative abundance (%)
*Lavandula stoechas*	12.79±22.25
*Helichrysum italicum*	25.62±32.20
*Achillea ligustica*	34.83±33.23
*Orlaya daucoides*	2.98±12.98
*Pulicaria odora*	11.77±21.59
*Stachys glutinosa*	1.97±7.39
*Teucrium capitatum*	2.71±14.44
*Phagnalon saxatile*	1.46±6.78
*Hedera helix*	1.02±8.69
*Vitis vinifera*	1.58±5.99
*Carduus sp.*	0.16±1.18
*Myrtus communis*	0.04±0.41
*Geranium robertianum*	0.53±4.99
*Cistus monspeliensis.*	1.09±5.82
*Foeniculum vulgare*	1.45±7.02

### Temporal variation in the use of aromatic plant species

#### a) Individual repeatability during the breeding season

In 2006, aromatic plant fragments were experimentally removed from 14 nests in the Muro sites (Muro-deciduous: 8 nests; Muro-evergreen: 6 nests), then collected again 24 h later. Plant fragments were sorted by species, allowed to dry in paper bags and weighed. This sampling was done at three different times in each nest (egg laying, incubation and chick rearing). To investigate whether composition in aromatic plant species is repeatable across breeding stages, we tested whether similarity in nest composition was higher within than among nests. Complementarily, we tested whether similarity was higher within than among breeding stages (egg-laying, incubation, chick rearing) (see below for details on similarity analyses).

#### b) Individual repeatability across years

We examined inter-year variation in aromatic plant species composition in nests of 27 females that bred at least twice between 2005 and 2007. Aromatic plants were sampled at days 14–15 post-hatching (see above). To investigate whether nest aromatic composition is repeatable across years, we tested whether similarity in nest composition was higher within than among individual females. Complementarily, we also tested whether similarity was higher within year than among years.

Similarities between pairs of nests were calculated using the Bray-Curtis index [35] on log-transformed relative abundances of aromatic plant species. Analyses of similarity were performed using the ANOSIM procedure, which is an approximate analogue of standard analysis of variance but based on similarity matrices [36]. Statistics are calculated from 999 pairwise permutations. These analyses were done with the Primer 6.1.6 software (Primer-E Ltd).

### Spatial and individual factors of variation among nests

Plant species composition of nests may depend on plant availability in the territory, female experience or female individual preferences (see Introduction). To test these three hypotheses, we compared similarities in plant species composition between pairs of nests built in different years, either among different breeding females in a given territory (i.e. nestbox) or among different territories for a given female. Comparisons of similarities were performed with t-tests in the R 2.6.0 software.

### Inter-site variation in the aromatic plant species composition of nests

To investigate inter-site variation in aromatic plant species composition, we performed a Principal Component Analysis on log-transformed relative abundances of aromatic plant species sampled from 115 nests over three consecutive years (2005: 62 nests, 2006: 25 nests, 2007: 28 nests). For those females that nested at least twice during the 3-year study period, we averaged the relative abundances of species over different years. The PC1, PC2 and PC3 scores accounted for 31.6%, 17.6% and 13.4% of variance, respectively. The effects of study site and year on PC1, PC2 and PC3 scores were tested with a multivariate analysis of variance (MANOVA) using the Wilks' lambda test in the R 2.6.0 software.

## Supporting Information

Table S1 Relative abundances in nests, and presence in the environment, of the five plant species that differ most between the “ARI” and “FIL” study plots. The presence or absence of plants in the study sites was assessed by a botanical specialist (A. Royaud) in the whole area covering each plot. Species saturation curves obtained during sampling indicated that sampling effort was appropriate (C. Petit, unpublished data). “% contrib. dissim.” is the relative contribution of each plant species to dissimilarity between plots (up to 90% dissimilarity), as calculated by the “Simper” procedure in the Primer 6.1.6 software.
(0.03 MB DOC)

Table S2 Juvenile dispersal between “ARI”, “FIL” and “GRA” study plots in the evergreen habitat. Number of juveniles that were recruited as breeders in the three study plots over the 2005–2007 study period. No adult dispersed between these sites after the first reproductive attempt. Total numbers of breeding individuals are indicated in parenthesis.
(0.02 MB DOC)

Figure S1 Variation in the aromatic plant species composition of nests according to the breeding plot. Axes are the same as those on Figure 3; they result from a PCA on the log-transformed relative abundances of 15 aromatic plant species (see Methods).
(6.84 MB TIF)

Figure S2 Variation in the aromatic plant species composition of nests according to the plot where females were born. Axes are the same as those on Figure 3; they result from a PCA on the log-transformed relative abundances of 15 aromatic plant species (see Methods).
(7.18 MB TIF)
