# Supplementary material for: Local Individual Preferences for Nest Materials in a Passerine Bird
Source: PLoS One. 2009 Apr 1;4(4):e5104. doi: 10.1371/journal.pone.0005104 (PMC2659446; doi:10.1371/journal.pone.0005104)
Supplement: Table S1 — Relative abundances in nests, and presence in the environment, of the five plant species that differ most between the “ARI” and “FIL” study plots. The presence or absence of plants in the study sites was assessed by a botanical specialist (A. Royaud) in the whole area covering each plot. Species saturation curves obtained during sampling indicated that sampling effort was appropriate (C. Petit, unpublished data). “% contrib. dissim.” is the relative contribution of each plant species to dissimilarity between plots (up to 90% dissimilarity), as calculated by the “Simper” procedure in the Primer 6.1.6 software. (0.03 MB DOC) [file pone.0005104.s001.doc]

**Table S1. Relative abundances in nests, and presence in the environment, of the five plant species that differ most between the “ARI” and “FIL” study plots.**

The presence or absence of plants in the study sites was assessed by a botanical specialist (A. Royaud) in the whole area covering each plot. Species saturation curves obtained during sampling indicated that sampling effort was appropriate (C. Petit, unpublished data). “% contrib. dissim.” is the relative contribution of each plant species to dissimilarity between plots (up to 90 % dissimilarity), as calculated by the “Simper” procedure in the Primer 6.1.6 software.

|  | Relative abundance in nests  (mean ± SE) | | Presence in  study plots | | % contrib. dissim. |
| --- | --- | --- | --- | --- | --- |
|  | Arinelle | Filagna | Arinelle | Filagna |
| *Lavandula stoechas* | 54.9 ± 11.7 | 3.1 ± 2.0 | × |  | 31.7 |
| *Helichrysum italicum* | 39.6 ± 9.6 | 22.6 ± 9.2 |  | × | 23.5 |
| *Achillea ligustica* | 4.8 ± 4.8 | 38.4 ± 9.6 |  |  | 22.9 |
| *Teucrium capitatum* | 0 ± 0 | 12.7 ± 8.7 |  |  | 7.7 |
| *Foeniculum vulgaris* | 0 ± 0 | 7.9 ± 4.5 | × | × | 4.8 |
