# Supplementary material for: Local Individual Preferences for Nest Materials in a Passerine Bird
Source: PLoS One. 2009 Apr 1;4(4):e5104. doi: 10.1371/journal.pone.0005104 (PMC2659446; doi:10.1371/journal.pone.0005104)
Supplement: Table S2 — Juvenile dispersal between “ARI”, “FIL” and “GRA” study plots in the evergreen habitat. Number of juveniles that were recruited as breeders in the three study plots over the 2005–2007 study period. No adult dispersed between these sites after the first reproductive attempt. Total numbers of breeding individuals are indicated in parenthesis. (0.02 MB DOC) [file pone.0005104.s002.doc]

**Table S2. Juvenile dispersal between ARI, FIL and GRA study plots in the evergreen habitat.**

Number of juveniles that were recruited as breeders in the three study plots over the 2005-2007 study period. No adult dispersed between these sites after the first reproductive attempt. Total numbers of breeding individuals are indicated in parenthesis.

|  | **Site of birth** | | |
| --- | --- | --- | --- |
| **Site of reproduction** | *ARI* | *FIL* | *GRA* |
| *ARI (45)* | 6 | 6 | 2 |
| *FIL (43)* | 4 | 4 | 2 |
| *GRA (74)* | 3 | 4 | 10 |
