# Supplementary figures and images for: Local Individual Preferences for Nest Materials in a Passerine Bird
Source: PLoS One. 2009 Apr 1;4(4):e5104. doi: 10.1371/journal.pone.0005104 (PMC2659446; doi:10.1371/journal.pone.0005104)

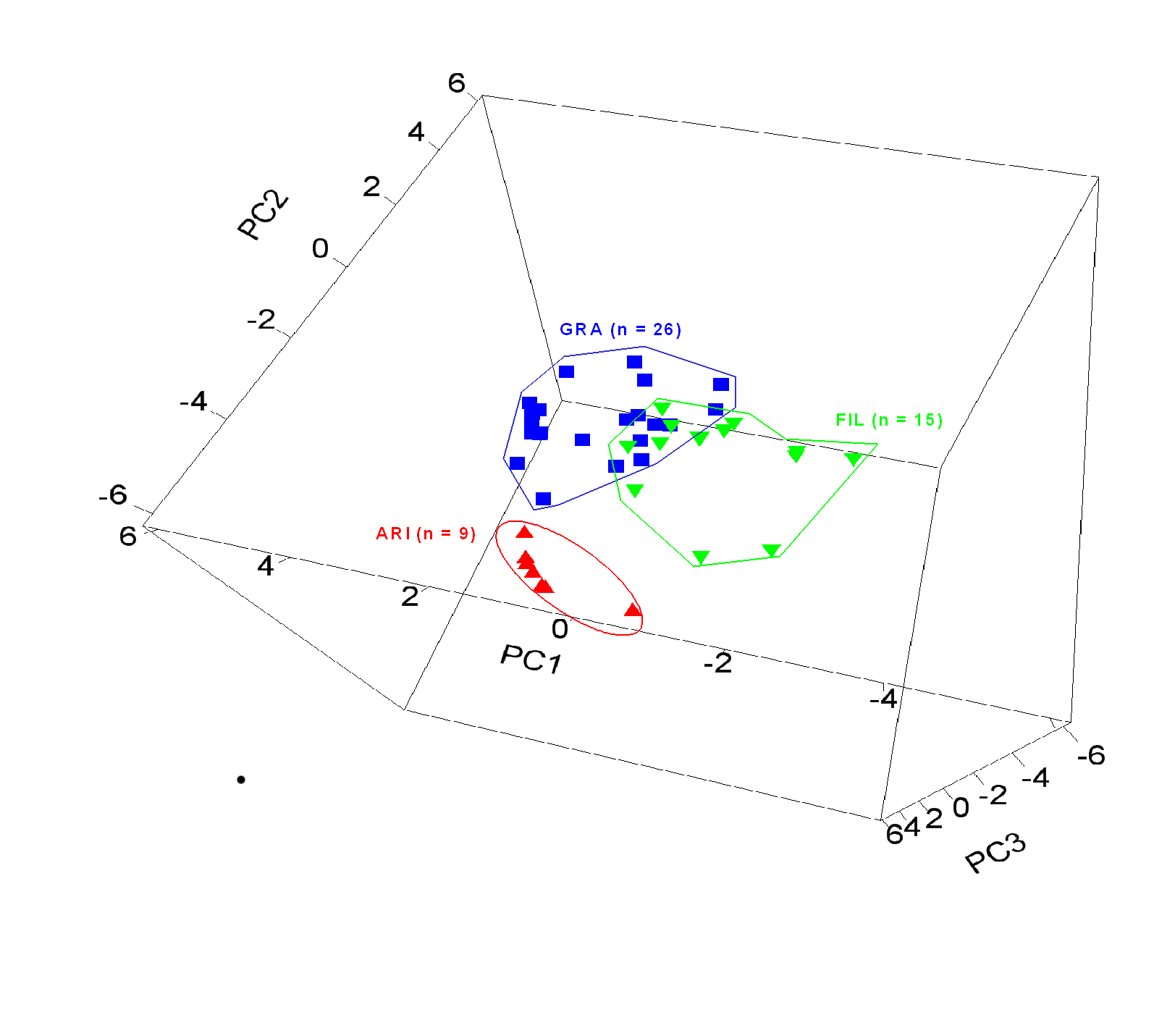

Supplement: Figure S1 — Variation in the aromatic plant species composition of nests according to the breeding plot. Axes are the same as those on Figure 3; they result from a PCA on the log-transformed relative abundances of 15 aromatic plant species (see Methods). (6.84 MB TIF) [file pone.0005104.s003.tif]

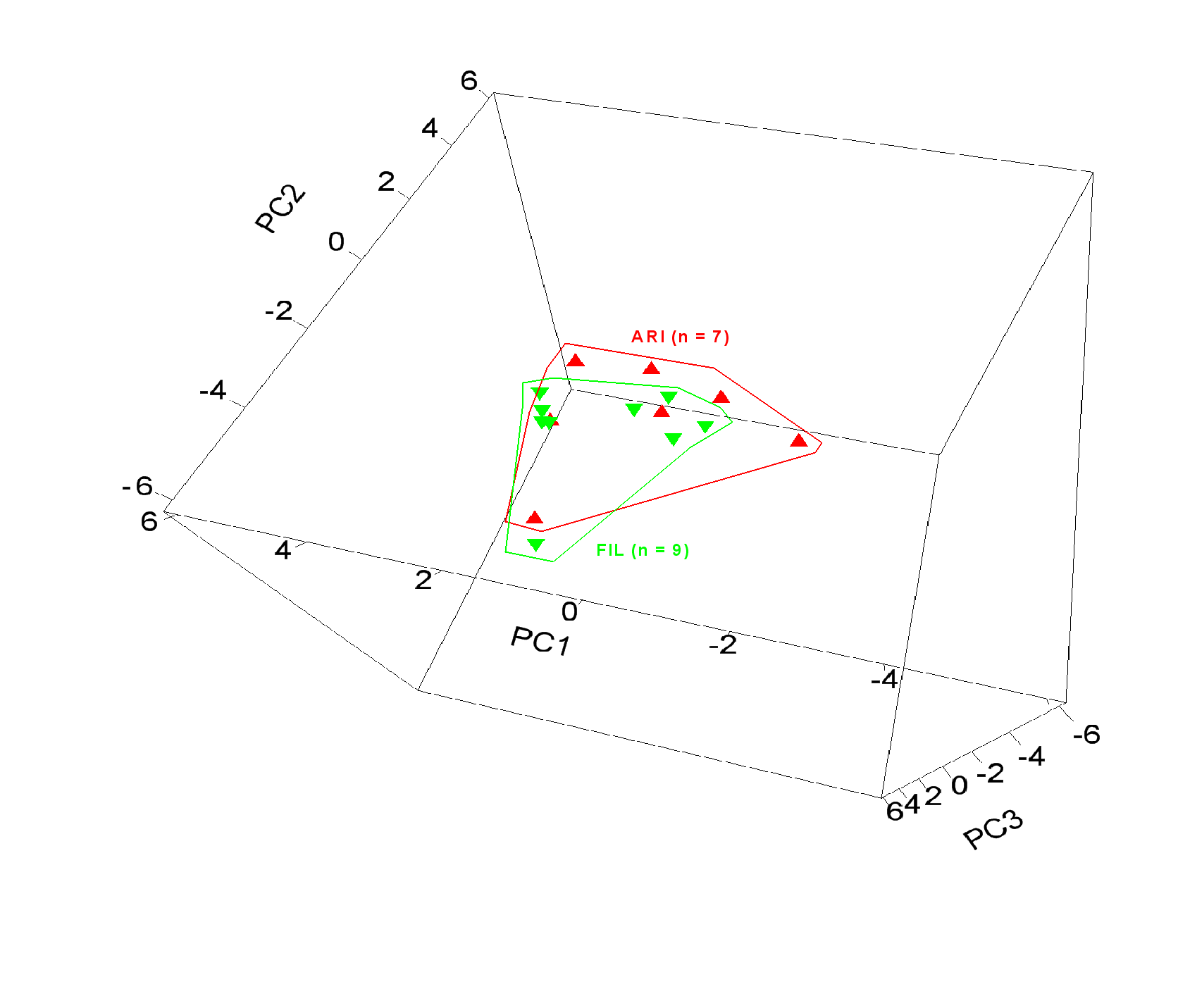

Supplement: Figure S2 — Variation in the aromatic plant species composition of nests according to the plot where females were born. Axes are the same as those on Figure 3; they result from a PCA on the log-transformed relative abundances of 15 aromatic plant species (see Methods). (7.18 MB TIF) [file pone.0005104.s004.tif]
